# Vitamin D Signaling from Nephrogenesis to Neoplasia: Spatial Protein Expression in Fetal Kidney and Transcriptomic Dysregulation in Renal Tumors

**DOI:** 10.3390/medicina62061074

**Published:** 2026-06-01

**Authors:** Ivana Bevanda, Natalija Filipović, Nela Kelam, Anita Racetin, Petar Todorović, Katarina Vukojević

**Affiliations:** 1 Department of Endocrinology, University Hospital Mostar, Bijeli Brijeg bb, 88000 Mostar, Bosnia and Herzegovina; bjelanovic.ivanaaa@yahoo.com; 2 Laboratory for Early Human Development, Department of Anatomy, Histology and Embryology, University of Split School of Medicine, Šoltanska 2A, 21000 Split, Croatia; natalija.filipovic@mefst.hr (N.F.); nela.kelam@mefst.hr (N.K.); anita.racetin@mefst.hr (A.R.); petar.todorovic@mefst.hr (P.T.); 3 Center for Translational Research in Biomedicine, University of Split School of Medicine, Šoltanska 2A, 21000 Split, Croatia; 4 Mediterranean Institute for Life Sciences (MedILS), University of Split, Meštrovićevo Šetalište 45, 21000 Split, Croatia

**Keywords:** vitamin D receptor, *VDR*, *CYP24A1*, *CYP27B1*, 1α-hydroxylase, kidney development, nephrogenesis, immunohistochemistry

## Abstract

*Background and Objectives*: Vitamin D signaling plays critical roles in immune regulation, bone metabolism, and cellular differentiation across multiple tissues. However, the spatial and temporal expression patterns of key vitamin D signaling components—the vitamin D receptor (VDR) and the enzyme 1α-hydroxylase (encoded by *CYP27B1*)—during human nephrogenesis have not been mapped at the protein level. The primary objective of this study was to characterize VDR and 1α-hydroxylase expression across critical stages of human kidney development, complementing prior transcriptomic and single-cell descriptions, and to contextualize these developmental observations against the dysregulation of vitamin D pathway genes in adult renal and urothelial malignancies. *Materials and Methods*: Immunofluorescence analysis was performed on FFPE kidney tissue from 12 specimens (3 per stage) at 10, 22 and 38 gestational weeks and postnatally at 1.5 years. For each specimen, at least three non-adjacent sections were stained and 6 non-overlapping cortical fields were imaged at ×40 (18 fields per stage). Fluorescence-area percentages were quantified in ImageJ 1.54g, and group differences were assessed by one-way ANOVA with Tukey’s post hoc test at both field- and specimen-level. An accompanying bioinformatic analysis evaluated the differential expression of *VDR*, *CYP27B1*, and *CYP24A1* in adult renal and urothelial malignancies (TCGA cohorts: KICH, KIRC, KIRP, BLCA) using unpaired Welch’s t-test, with Benjamini–Hochberg FDR correction applied across all 16 tumor-versus-normal comparisons (12 gene-wise + 4 post hoc log_2_(CYP24A1/CYP27B1) ratios). *Results*: VDR showed its highest mean fluorescence area at 10 weeks (3.40% (95% CI 3.24–3.56); field-level Tukey *p* < 0.0001 versus other stages) and its lowest at 22 weeks (0.69% (0.64–0.74)). 1α-hydroxylase was also highest at 10 weeks (5.44% (5.29–5.60); *p* < 0.0001) and stabilized at lower levels thereafter (3.04–4.26%). Co-expression of both proteins was observed throughout development except in 22-week glomeruli. In TCGA, all 12 significant gene-wise comparisons retained significance after BH FDR correction (q < 0.05). VDR showed cohort-specific dysregulation: reduced in KICH (q = 2 × 10^−4^) but increased in KIRC and KIRP (q = 2 × 10^−4^ for both). CYP24A1 was reduced in all three renal cohorts (q ≤ 0.029) and unchanged in BLCA. CYP27B1 showed cohort-specific direction (reduced in KIRC; increased in KICH, KIRP, and BLCA). *Conclusions*: This study provides an initial immunofluorescence-based spatial description of VDR and 1α-hydroxylase across human kidney development, revealing a coordinated redistribution from immature glomeruli at 10 weeks to mature tubular segments at later stages. The TCGA analysis demonstrates that vitamin D pathway dysregulation in renal carcinoma is cohort-specific and is not abolished by multiple-testing correction. Together, these results indicate that the developmentally engaged vitamin D pathway retains kidney-specific functional relevance in adult renal pathology and provide a baseline reference for future mechanistic studies.

## 1. Introduction

The formation of the human kidney is a complex morphogenetic process driven by reciprocal signaling between epithelial and mesenchymal cell populations, which together orchestrate the growth and specification of functionally distinct renal cell lineages [1]. During normal renal morphogenesis, signals from the ureteric bud induce the surrounding metanephric mesenchyme to condense and form cap mesenchyme aggregates [2]. Nephron progenitors then advance through a series of morphologically defined intermediates—renal vesicle, comma-shaped body, and S-shaped body—before maturing into fully formed nephrons and glomeruli [3,4,5], structures indispensable for physiological renal function. However, the involvement of the vitamin D receptor (VDR) and 25-hydroxyvitamin D3-alpha-hydroxylase (1α-hydroxylase), a key enzyme in vitamin D metabolism, remains unknown in kidney development. Namely, several reports provide evidence that vitamin D deficiency plays a role in the development of kidney diseases [6,7], but its involvement in normal kidney development remains unexplored. Prior transcriptomic work has documented expression of vitamin D-metabolizing enzymes in the first trimester [8] and single-cell transcriptomic atlases of the human fetal kidney are now available [9]; however, systematic immunofluorescence-based spatial mapping of VDR and CYP27B1 protein localization across the full course of human nephrogenesis has not been reported.

VDR, also known as NR1I1, mediates the genomic actions of vitamin D [10]. VDR is a ligand-activated transcription factor belonging to the nuclear receptor (NR) superfamily [11]. It regulates the expression of numerous genes primarily involved in calcium-phosphate balance and bone metabolism [12,13]. In the adult kidney, VDR is found in the epithelial cells of the proximal and distal tubules, the collecting duct epithelial cells, and the podocytes [14]. The hormonally active metabolite 1α,25(OH)_2_D_3_ represents the highest-affinity natural VDR ligand and additionally modulates cell proliferation, differentiation, programmed cell death, and immune function [12,13,15].

The vitamin D metabolic pathway involves two key enzymes: *CYP27B1* (1α-hydroxylase) which activates 25(OH)D_3_ to the bioactive 1α,25(OH)_2_D_3_, and *CYP24A1* (24-hydroxylase) which inactivates it [6,15,16]. The expression and activity of 1α-hydroxylase are regulated by parathyroid hormone (PTH), fibroblast growth factor 23 (FGF23), and 1α,25(OH)_2_D_3_ [17]. The enzyme is primarily expressed in the kidneys but is also found in various non-renal tissues, including the skin [18,19,20]. Notably, *CYP24A1* loss-of-function mutations cause idiopathic infantile hypercalcemia [21,22], underscoring its developmental clinical relevance.

Despite extensive characterization of vitamin D signaling in adult renal physiology and disease, three specific knowledge gaps remain. First, although prior transcriptomic studies have detected vitamin D-metabolizing enzyme transcripts in the first-trimester human kidney [8,23], and single-cell atlases now describe the cellular composition of the human fetal kidney [9], no study has mapped the spatial distribution of VDR or 1α-hydroxylase at the protein level across the full course of human nephrogenesis. Second, it is not known whether the VDR and 1α-hydroxylase that are robustly expressed in the adult nephron appear early enough during development to participate in nephron induction and maturation, or only later as the tubular machinery becomes functionally competent. Third, the question of whether vitamin D pathway genes engaged during nephrogenesis retain functional relevance in adult renal tissue—and become dysregulated in renal pathology—has not been examined in a developmentally informed framework.

Addressing these gaps is clinically relevant because perturbations of vitamin D signaling during kidney development could plausibly contribute to congenital anomalies of the kidney and urinary tract (CAKUT) and to lifelong susceptibility to renal disease, yet this hypothesis cannot be tested without baseline knowledge of when and where vitamin D pathway components are expressed in the developing human kidney.

We therefore designed the present study with two complementary objectives: to characterize the spatial and temporal expression of VDR and 1α-hydroxylase across critical stages of human kidney development (10, 22, and 38 gestational weeks and postnatal kidney at 1.5 years) by quantitative immunofluorescence, and to contextualize these developmental observations against the dysregulation of vitamin D pathway genes (*VDR*, *CYP27B1*, *CYP24A1*) in adult renal and urothelial malignancies (TCGA cohorts: KICH, KIRC, KIRP, BLCA). Together, these two analyses were intended to provide an initial developmental reference framework for vitamin D signaling in the human kidney and to assess whether the same pathway components remain functionally relevant in adult renal pathology.

## 2. Materials and Methods

### 2.1. Tissue Collection and Processing

Tissue specimens were obtained from the Departments of Gynaecology/Obstetrics and Pathology following spontaneous pregnancy losses or ectopic (tubal) pregnancies. A total of 12 specimens (9 fetal, 3 postnatal) were collected and handled in accordance with the Declaration of Helsinki, with approval from the Ethics and Drug Committee of University Hospital Split. Conceptus age was determined on the basis of menstrual history, crown–rump length measurements, and Carnegie stage criteria. Three tissue specimens per developmental stage were included: three at 10 weeks, three at 22 weeks, three at 38 weeks, and three postnatal specimens from previously healthy children aged approximately 1.5 years. The parents previously signed informed consent for this research. All tissues showed no signs of maceration and appeared morphologically normal. Specimens were immersed in 4% paraformaldehyde/PBS, processed through a graded ethanol series, embedded in paraffin, and cut at 5 µm on a rotary microtome (RM2125 RTS; Leica, Buffalo Grove, IL, USA). Adequate preservation was confirmed by hematoxylin–eosin (H&E) staining of every tenth section, examined on an Olympus BX51 microscope (Olympus, Tokyo, Japan).

### 2.2. Immunohistochemistry and Immunofluorescence Staining

Sections were deparaffinized and rehydrated through descending ethanol concentrations according to an established protocol [24,25,26]. Heat-mediated antigen retrieval was carried out in 0.01 M sodium citrate buffer (pH 6.0). Following PBS washes, non-specific binding was blocked with Protein Block reagent (ab64226, Abcam, Cambridge, UK). Sections were then incubated overnight with primary antibodies (Table 1), rinsed in PBS, and exposed to the corresponding secondary antibodies (Table 1) for 1 h in a humidified chamber.

After additional PBS rinses, nuclei were counterstained with 4′,6-diamidino-2-phenylindole (DAPI), and coverslips were mounted. Two negative-control strategies were employed to minimize non-specific background: isotype-matched controls (primary antibody replaced by an irrelevant antibody of identical isotype) and secondary-only controls (primary antibody omitted). Together, these controls verified that fluorescence signals reflected genuine target-protein expression rather than artefactual background staining. Representative secondary-only negative control images for each developmental stage (10 weeks, 22 weeks, 38 weeks, and 1.5 years) are provided in Supplementary Figure S1; these controls were imaged under identical exposure and gain settings as the corresponding primary-stained sections and were used to calibrate the background threshold applied during ImageJ quantification (Section 2.3).

### 2.3. Data Collection and Image Analysis

Images were acquired on an Olympus BX51 epifluorescence microscope equipped with a Nikon DS-Ri2 digital camera (Nikon Corporation, Tokyo, Japan) running NIS-Elements F software (v 5.22.00). For each of the 12 specimens (3 per developmental stage), at least three non-adjacent paraffin sections were stained, and 6 non-overlapping cortical fields per specimen were captured at ×40 magnification under uniform gain and exposure conditions, yielding 18 individual field measurements per developmental stage (3 specimens × 6 fields). The complete list of individual field-level values is provided in Supplementary Table S1.

Quantitative image analysis followed published protocols [27,28,29] and was carried out in ImageJ (v 1.54; NIH, Bethesda, MD, USA). In brief, the red counter-channel was subtracted from the green channel to reduce spectral leakage. Each image was duplicated; one copy underwent median filtering (radius 8.0 px) and was then subtracted from the unfiltered duplicate to isolate the positive signal. After conversion to 8-bit grayscale, the triangle auto-threshold was applied, and the percentage of fluorescent area was obtained via the “analyze particles” function. Three experienced histologists scored all micrographs independently, calibrating background thresholds against the matched secondary-only negative control images (Supplementary Figure S1) acquired under identical optical settings. Interclass correlation analysis yielded a coefficient > 0.8, confirming excellent inter-rater agreement [30].

Structural assignment of immature glomeruli, proximal convoluted tubules (PCT), distal convoluted tubules (DCT) and collecting ducts was performed independently and in consensus by two senior renal histopathologists with extensive experience in human kidney development, with disagreements resolved by joint re-evaluation of the original DAPI/H&E sections at high magnification. We emphasize that these assignments rest on histological criteria alone; segment-specific lineage markers were not co-stained in the present study. Compartment identification is therefore subject to interpretive variability, particularly at early developmental stages where nephron segments are not yet fully differentiated; this is acknowledged explicitly in the Limitations section.

### 2.4. Statistical Analysis

Data handling was performed in Excel v2403 (Microsoft Corporation, Redmond, WA, USA); inferential statistics are described below (last paragraph of this Section). Significance was set at *p* < 0.05. Data normality was verified with the Shapiro–Wilk test. Fluorescence area percentages were compared across developmental stages by one-way ANOVA with Tukey’s post hoc correction for multiple comparisons. Values are reported as mean and 95% confidence interval (CI); standard deviations and exact *p*-values are provided in Supplementary Table S1. 

Because each specimen contributes multiple fields, both levels of analysis were performed. Field-level Tukey HSD was applied to all 18 fields per stage, treating individual fields as separate observations, while specimen-level Tukey HSD was performed on the three specimen means per stage. The field-level approach assumes independence of fields, which does not strictly hold, whereas the specimen-level approach avoids pseudoreplication but is limited by the small sample size (*n* = 3 per stage). Both analyses are reported together to allow direct comparison of their outcomes.

Bar graphs displaying mean fluorescence area percentages with overlaid scatter points were generated in Python 3.11 using matplotlib (v3.8) and pandas (v2.1), with statistical analysis performed in SciPy (v1.11; scipy.stats.tukey_hsd for Tukey’s honestly significant difference post hoc tests and scipy.stats.f_oneway for one-way ANOVA). TCGA-derived gene expression box plots were generated in R using the ggplot2 package (v4.0.2). Final figure assembly was performed in Adobe Photoshop CS2 (v9.0). Background reduction and contrast enhancement were applied to the microphotographs to improve their presentation.

### 2.5. Semi-Quantification

Staining intensity was graded semi-quantitatively on a four-tier scale: no reactivity (−), weak (+), moderate (++), and strong (+++) (Table 2). Three investigators independently scored all captured images in ImageJ.

This semi-quantitative approach was retained for its utility in describing structure-specific localization; however, future studies should complement or replace it with direct, compartment-specific fluorescence intensity quantification in ImageJ to minimize inter-rater subjectivity.

### 2.6. In Silico Gene Expression Analysis

To contextualize our developmental findings within the broader framework of renal pathology, we performed a secondary in silico analysis of gene expression in adult kidney tissue. Transcript-level data for *VDR*, *CYP24A1* and *CYP27B1* were retrieved from the publicly accessible RNA-seq repository of The Cancer Genome Atlas (TCGA; last accessed 10 December 2025), using harmonized RSEM-normalized TPM values via cBioPortal. We analyzed four cohorts: chromophobe renal cell carcinoma (KICH; 25 solid-tissue-normal/66 tumor), clear cell renal cell carcinoma (KIRC; 72/533), papillary renal cell carcinoma (KIRP; 32/290) and urothelial carcinoma of the bladder (BLCA; 19/408). For each gene in each cohort, tumor and solid-tissue-normal samples were compared with an unpaired two-tailed Welch’s *t*-test. These twelve gene-wise comparisons were pre-specified as the primary inferential analysis of the TCGA component of the study.

As an additional analysis, the metabolic balance between vitamin D activation and inactivation was assessed by computing the log_2_((*CYP24A1* + 1)/(*CYP27B1* + 1)) ratio per sample and comparing the resulting per-sample ratios between tumor and normal in each cohort (four additional comparisons). This ratio analysis is designated post hoc and exploratory, as it was undertaken after inspection of the gene-wise results.

To control family-wise type-I error inflation across the full panel of sixteen tumor-versus-normal tests (12 gene-wise + 4 ratio), all *p*-values were jointly corrected for multiple testing using the Benjamini–Hochberg false discovery rate (FDR) procedure, treating the sixteen tests as a single comparison family. Both raw *p*-values and BH-adjusted q-values are reported in the Results Section, and in Supplementary Table S2. Plots were created in R using the ggplot2 package (v 4.0.2).

## 3. Results

### 3.1. H&E Staining

**Figure 1:**
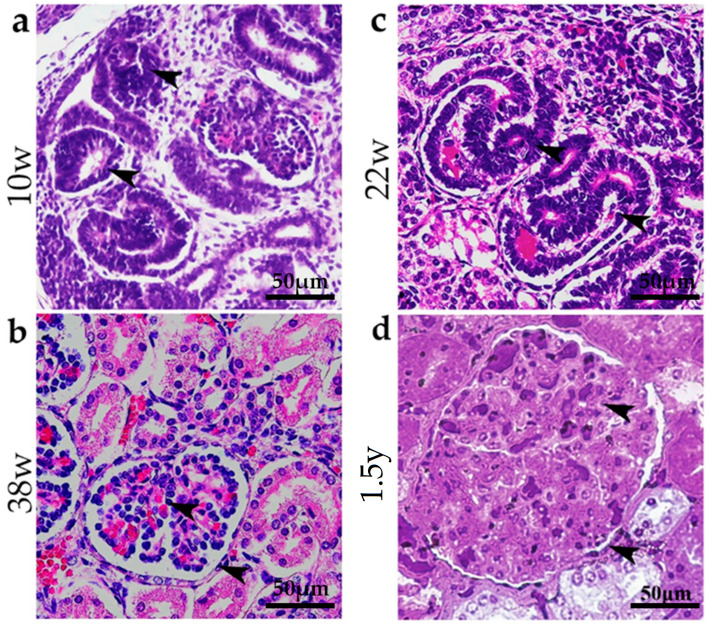
Human kidney in the 10th week of development with renal vesicles (arrows, panel (**a**)); 22nd week of development with S-shaped nephrons (arrows, panel (**b**)); 38th week of development with a glomerulus surrounded by Bowman’s capsule (arrows, panel (**c**)); 1.5 years postnatal sample with mature glomerulus surrounded by Bowman’s capsule (arrows, panel (**d**)).

In the earliest developmental window examined, inductive signals from the ureteric bud prompt metanephric cap mesenchyme to undergo epithelialization, generating the lumen-containing renal vesicle (Figure 1a)—a transformation known as mesenchymal–epithelial transition. With continued maturation, the vesicle remodels into an S-shaped body (Figure 1b) that subsequently gives rise to nascent glomeruli (Figure 1c) and the tubular segments of the nephron (Figure 1d).

### 3.2. Immunofluorescence Staining with VDR and 1α-Hydroxylase

The spatial distribution of VDR and 1α-hydroxylase was examined in developing and postnatal human kidney tissue.

**Figure 2:**
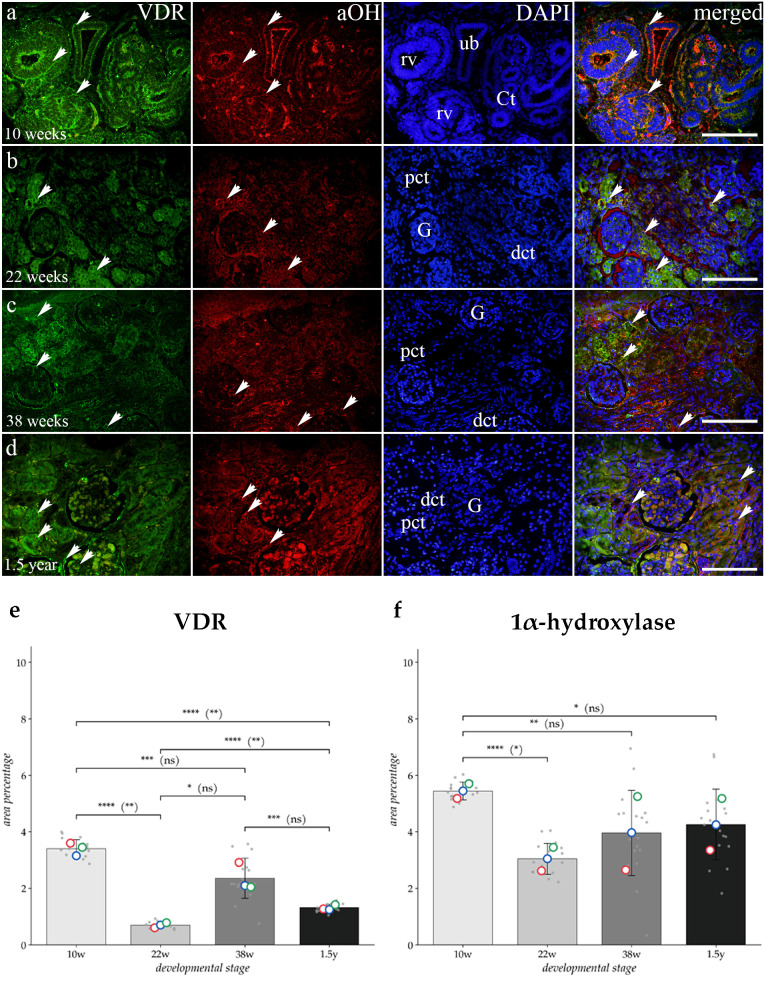
Immunoexpression of renal vitamin D receptors (VDR) and 25-hydroxy-vitamin D3-alpha-hydroxylase (1α-hydroxylase) in the developing and postnatal human kidneys. Representative micrographs at 10 gestational weeks (**a**), 22 weeks (**b**), 38 weeks (**c**) and 1.5 years postnatal (**d**). Arrows indicate the expression pattern of VDR and 1α-hydroxylase in the ureteric bud (ub), collecting tubule (Ct), renal vesicle (rv), glomeruli (G), proximal convoluted (pct), and distal convoluted tubules (dct) as indicated in the 4′,6-diamidino-2-phenylindole (DAPI) image. ×40 magnification; scale bar = 100 µm (applies to all panels). Quantification of VDR (**e**) and 1α-hydroxylase (**f**) fluorescence area percentage across developmental stages. Small grey dots show all 18 individual field measurements per stage (3 specimens × 6 non-overlapping cortical fields per specimen); large colored open circles show the three per-specimen means; bars show stage mean ± SD. Two levels of analysis are shown on every bracket: the symbol above the bracket reports the field-level Tukey HSD *p*-value (*n* = 18 per stage), and the symbol in parentheses reports the specimen-level Tukey HSD *p*-value (*n* = 3 per stage). Significance codes: **** *p* < 0.0001, *** *p* < 0.001, ** *p* < 0.01, * *p* < 0.05, ns = not significant. Individual values used to compute these summaries are provided in Supplementary Table S1.

During the 10th week of development, the VDR was strongly expressed in immature glomeruli, moderately expressed in the metanephric cup, and mildly expressed in the collecting tubules (Figure 2a, Table 2). By the 22nd week of development, VDR showed moderate expression in both proximal and distal convoluted tubules, with mild expression in the glomeruli (Figure 2b, Table 2). In the 38th week of development, VDR maintained moderate expression in the proximal convoluted tubules and mild expression in the glomeruli and distal convoluted tubules (Figure 2c, Table 2). In postnatal kidney tissue (1.5 years), VDR expression remained consistent with the 22nd week of development, showing moderate expression in the proximal and distal convoluted tubules and mild expression in the glomeruli (Figure 2d, Table 2). At the 10th week of development, 1α-hydroxylase was moderately expressed in the metanephric cup, mildly expressed in the collecting tubules, and found in immature glomeruli (Figure 2a, Table 2). By the 22nd week of kidney development, 1α-hydroxylase showed moderate expression in the proximal and distal convoluted tubules, as well as in the glomeruli (Figure 2b, Table 2). At the 38th week, 1α-hydroxylase expression remained moderate in the distal convoluted tubules and glomeruli, while being mildly expressed in the proximal tubules (Figure 2c, Table 2). At 1.5 years postnatally, 1α-hydroxylase showed comparable expression levels across proximal tubules, distal tubules, and glomeruli (Figure 2d, Table 2).

Co-expression of VDR and 1α-hydroxylase was observed in all stages (from 10th week to 1.5 years) and in all kidney structures, except in the glomeruli of the 22nd week of development (Figure 2a–d).

The area percentage of VDR at the 10th week of development was 3.40%, which was the highest value and significantly different from the other time points (*p* < 0.001 and *p* < 0.0001, respectively) (Figure 2e). In contrast, the VDR area percentage at the 22nd week was the lowest at 0.69%. This value also showed a statistically significant difference compared to the other periods (*p* < 0.05 and *p* < 0.0001, respectively) (Figure 2e).

The area percentage of 1α-hydroxylase in the 10th week of development was 5.44%. This value was the highest, and this difference was statistically significant compared with the other investigated periods (*p* < 0.05, *p* < 0.01, and *p* < 0.0001, respectively; Figure 2f). After the 22nd week, the area percentage of 1α-hydroxylase remained lower than at the 10th week, ranging from 3.04% to 4.26% across the remaining stages, without statistically significant differences among the 22nd week, 38th week, and 1.5-year time points.

### 3.3. In Silico Analysis of Vitamin D-Related Gene Expression in Adult Renal Tissues

**Figure 3:**
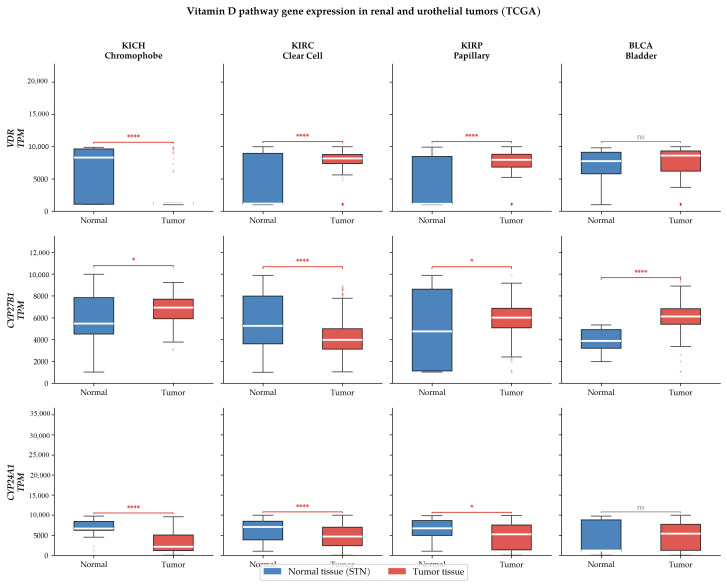
Tissue-specific dysregulation of vitamin D signaling genes in renal and urothelial tumors. RNA-sequencing-derived expression levels of *VDR, CYP27B1,* and *CYP24A1* in solid tissue normal (STN) controls and corresponding tumor samples from four TCGA cohorts: kidney chromophobe carcinoma (KICH, *n* = 25 normal/66 tumor), kidney renal clear cell carcinoma (KIRC, *n* = 72 normal/533 tumor), kidney renal papillary cell carcinoma (KIRP, *n* = 32 normal/290 tumor), and bladder urothelial carcinoma (BLCA, *n* = 19 normal/408 tumor). Gene expression is displayed as transcripts per million (TPM). Tumor-versus-normal comparisons were performed by unpaired two-tailed Welch’s *t*-test (pre-specified primary analysis); raw *p*-values are reported in the figure, and Benjamini–Hochberg FDR-adjusted q-values across all 16 tumor-versus-normal comparisons (12 gene-wise + 4 log_2_-ratio) are reported in Supplementary Table S2. **** *p* < 0.0001, * *p* < 0.05, ns = not significant; all significant comparisons retained significance after BH FDR correction.

To examine whether the spatiotemporal expression patterns identified during development are reflected in tissue-specific dysregulation in adult renal pathology, we compared *VDR* (Figure 3), *CYP27B1* (Figure 3), and *CYP24A1* (Figure 3) expression between tumor and adjacent normal samples in each of the four TCGA cohorts. Benjamini–Hochberg FDR correction was applied across all sixteen comparisons (12 gene-wise + 4 ratio); all twelve gene-wise comparisons reaching nominal significance at raw *p* < 0.05 retained significance after correction (Supplementary Table S2).

*VDR* shows cohort-specific, opposite-direction dysregulation across renal carcinoma subtypes. In KICH, *VDR* expression was significantly reduced in tumor versus adjacent normal tissue (mean TPM 6.47 versus 2.65; log_2_FC = −1.03; q = 2 × 10^−4^), consistent with profound loss of vitamin D receptor signaling capacity in chromophobe carcinoma. By contrast, *VDR* was significantly increased in tumor versus normal in both KIRC (4.79 versus 7.75; log_2_FC = +0.59; q = 2 × 10^−4^) and KIRP (4.04 versus 7.37; log_2_FC = +0.73; q = 2 × 10^−4^). No significant difference was observed in BLCA (q = 0.97), consistent with kidney-restricted rather than urological-wide *VDR* dysregulation. The directional divergence between KICH and the other two renal carcinoma subtypes is biologically notable and is discussed below.

*CYP27B1* also shows cohort-specific direction. *CYP27B1* was significantly reduced in KIRC (log_2_FC = −0.32; q = 2 × 10^−4^) but significantly increased in KICH (log_2_FC = +0.19; q = 0.035), KIRP (log_2_FC = +0.17; q = 0.029) and BLCA (log_2_FC = +0.62; q = 2 × 10^−4^). The cohort-specific direction in vitamin D activation capacity argues against a single uniform mechanism across renal cancer types and points toward subtype-specific reprogramming of the vitamin D activation axis.

*CYP24A1* is uniformly reduced across all three renal cohorts. *CYP24A1*, encoding the inactivating 24-hydroxylase, was significantly reduced in all three renal cohorts after FDR correction (KICH log_2_FC = −0.95, q = 2 × 10^−4^; KIRC log_2_FC = −0.31, q = 2 × 10^−4^; KIRP log_2_FC = −0.29, q = 0.029), with no significant change in BLCA (q = 0.24). Reduced CYP24A1 expression suggests diminished local vitamin D catabolic capacity in renal tumors; this is the single vitamin D pathway gene that shows a consistent direction of change across all three renal carcinoma subtypes.

**Figure 4:**
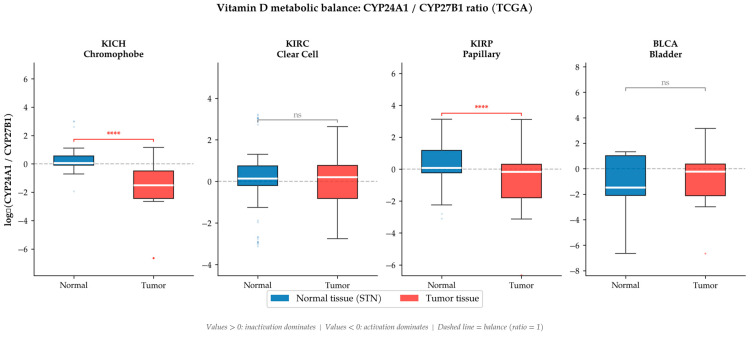
Vitamin D metabolic balance: log_2_(*CYP24A1*/*CYP27B1*) expression ratio across TCGA cohorts. Per-sample ratios were computed as log_2_((*CYP24A1* + 1)/(*CYP27B1* + 1)) and compared between solid-tissue-normal (blue) and tumor (red) groups in each cohort. Values above 0 indicate predominance of the inactivating enzyme; values below 0 indicate predominance of the activating enzyme; the dashed line at 0 represents metabolic balance. This ratio analysis was post hoc and exploratory and is reported as hypothesis-generating; raw *p*-values are shown in the figure and Benjamini–Hochberg FDR-adjusted q-values across all 16 comparisons (12 gene-wise + 4 ratio) are reported in Supplementary Table S2. **** *p* < 0.0001.

In the post hoc exploratory analysis of the log_2_(*CYP24A1/CYP27B1*) ratio (Figure 4), KICH showed a significantly decreased ratio in tumor versus normal (Δ = −1.51; raw *p* = 2 × 10^−9^; q = 3.5 × 10^−8^), driven principally by the strong CYP24A1 reduction. KIRP also showed a significant decrease (Δ = −0.77; q = 0.003). No significant ratio shift was observed in KIRC (q = 0.44)—in which *CYP24A1* and *CYP27B1* are reduced in parallel—or in BLCA (q = 0.44). Because the ratio analysis was post hoc, we interpret these results as hypothesis-generating rather than confirmatory.

Taken together, renal carcinomas (KICH, KIRC, KIRP) display dysregulation of all three vitamin D pathway genes, but the direction of *VDR* and *CYP27B1* change is cohort-specific rather than uniform; *CYP24A1* is consistently reduced across renal cohorts. BLCA shows isolated up-regulation of *CYP27B1* only. This pattern supports a kidney-specific biology of vitamin D pathway dysregulation while highlighting biologically meaningful heterogeneity among renal cancer subtypes.

## 4. Discussion

The present study was designed to address two related questions: whether the protein-level distribution of VDR and 1α-hydroxylase across human nephrogenesis is consistent with active local vitamin D signaling during kidney development, and whether the same pathway components remain functionally relevant in adult renal tissue, as suggested by their dysregulation in renal malignancy. Our data answer both questions in the affirmative and reveal three principal findings.

First, both VDR and 1α-hydroxylase show their highest fluorescence area at 10 gestational weeks (3.40% and 5.44%, respectively), declining at 22 weeks and partially recovering thereafter, with the principal pairwise differences supported at both the field- and specimen-level of analysis. Second, the spatial pattern of VDR expression undergoes a marked redistribution between early and late gestation: at 10 weeks, expression is concentrated in immature glomeruli and the metanephric cap, whereas by 22 weeks and beyond, it shifts to the proximal and distal convoluted tubules—a transition that parallels the functional maturation of nephron segments. VDR and 1α-hydroxylase co-localize across most developmental stages and nephron compartments, with the notable exception of glomeruli at 22 weeks. Third, the in silico TCGA analysis demonstrates that vitamin D pathway genes engaged during nephrogenesis are dysregulated in adult renal carcinomas in a cohort-specific manner that survives multiple-testing correction; *CYP24A1* is uniformly reduced across renal cohorts, while *VDR* and *CYP27B1* show opposite directions of change between chromophobe carcinoma and clear-cell/papillary subtypes. Together, these findings provide an initial spatial protein-level description of vitamin D signaling during human nephrogenesis, complementary to existing transcriptomic and single-cell evidence.

The remainder of the Discussion considers each of these findings in the context of the published literature [8,9,31]. At the 10th week of gestation, VDR expression was notably strong in immature glomeruli and less pronounced in other structures such as the metanephric cup and collecting tubules. By the 22nd week, VDR expression shifted toward the proximal and distal convoluted tubules, suggesting a functional transition as these structures mature and assume more defined roles in vitamin D metabolism and overall kidney function. This moderate expression pattern persisted into the 38th week and postnatal period (1.5 years), underscoring a sustained requirement for VDR in mature nephron segments. Although VDR distribution is well documented in the adult nephron—proximal and distal tubular epithelium, collecting ducts, and podocytes [14]—its developmental expression trajectory across human nephrogenesis had not been described before the present work. Our findings thus provide the first spatial protein-level evidence for this developmental pattern in human tissue.

Studies of VDR in other developmental and adult tissues provide context for interpreting our findings. Arora et al. identified VDR in hematopoietic progenitor cells and reported especially robust expression in neutrophils and monocytes, implying that immune cell populations respond directly to vitamin D [32]. Jelcic et al. revealed strong VDR expression in both stromal and trophoblastic components of the placenta, with weaker expression in preeclamptic tissue [33]. Song et al. showed that *VDR*-deficient retinal endothelial cells exhibit impaired proliferation, enhanced adhesion, and reduced migratory capabilities, along with heightened sensitivity to oxidative stress [34]. Together, these studies demonstrate that VDR plays tissue-specific roles in cellular differentiation, adhesion, and homeostasis across multiple organ systems, which is consistent with our observation of dynamic, structure-specific VDR expression in the developing kidney.

In parallel, the expression of 1α-hydroxylase exhibited distinct yet complementary patterns to those of VDR. High expression at the 10th week underscores its potential function in early kidney development, possibly facilitating local production of active vitamin D to regulate cellular processes. Statistical analysis revealed a significant decrease in fluorescence area from the 10th to the 22nd week (*p* < 0.05), with expression levels stabilizing thereafter—a pattern that may reflect tightly regulated adjustment of the local vitamin D synthesizing capacity as the kidney matures and systemic hormonal controls become more established. Our findings align with and build upon the murine data of Yamagata et al. [31], who showed that 1α-hydroxylase transcript appears in mouse kidney organ cultures between embryonic days 13.5 and 17.5 (approximately equivalent to human gestational weeks 6–8), with expression confined to the tubular epithelium of the developing kidney. Our findings provide the first complementary human protein-level data across a broader developmental window (10–38 weeks and postnatal). Earlier transcriptomic studies have confirmed the presence of vitamin D-metabolizing enzyme transcripts during the first trimester [8], and single-cell RNA-sequencing atlases of the human fetal kidney have since become openly accessible [9]. Zehnder et al. further confirmed the presence of 1α-hydroxylase in human endothelial cells, with rapid induction by inflammatory cytokines suggesting a novel autocrine/paracrine role [35]. Similar tissue-specific variations in 1α-hydroxylase expression have been reported in other organs, including differential expression between benign and malignant ovarian tissues [36]. However, none of these studies performed systematic immunofluorescence-based spatial mapping of VDR and CYP27B1 protein localization across the full course of human nephrogenesis, which is the primary contribution of the present work.

The co-expression of VDR and 1α-hydroxylase across all developmental stages and most kidney structures—except in the glomeruli at the 22nd week—supports the concept that the developing human kidney is capable of locally activating and responding to vitamin D in an autocrine and/or paracrine manner. This pattern also indicates tightly regulated, stage-specific roles rather than uniform signaling throughout development. Wang et al. showed that proximal tubular VDR monitors circulating 1α,25(OH)_2_D_3_ concentrations; upon ligand binding, the activated VDR–1α,25(OH)_2_D_3_ complex downregulates 1α-hydroxylase transcription in a negative-feedback loop [37]. Our developmental data suggest that this regulatory relationship may already be operational during nephrogenesis, potentially contributing to the structure-specific dissociation of co-expression observed at certain stages.

Our in silico TCGA analysis demonstrates that the vitamin D pathway genes engaged during nephrogenesis are dysregulated in adult renal carcinoma, and that the dysregulation is cohort-specific rather than uniform. After Benjamini–Hochberg correction across all sixteen tumor-versus-normal comparisons, twelve of twelve nominally significant gene-wise findings retained significance, indicating that the principal observations are robust to multiple-testing burden. *VDR* is significantly reduced in chromophobe renal cell carcinoma (KICH) but significantly increased in clear-cell (KIRC) and papillary (KIRP) renal cell carcinoma; *CYP27B1* follows the opposite direction in KIRC compared with the other three cohorts; and *CYP24A1* is significantly reduced in all three renal cohorts. Bladder urothelial carcinoma (BLCA) shows an isolated up-regulation of *CYP27B1* only, with no significant change in *VDR* or *CYP24A1*, supporting the interpretation that simultaneous dysregulation of all three pathway genes is a kidney-restricted feature rather than a generic feature of urological malignancy. These findings are consistent with the recent pan-cancer analysis of Xia et al. [38], who reported *VDR* down-regulation in KIRC and KIRP and up-regulation in KICH across the same TCGA cohorts.

The directional divergence of VDR dysregulation across renal subtypes is biologically interpretable in terms of cell-of-origin biology. KICH arises from the intercalated cells of the collecting duct; KIRC originates from cells with a proximal-tubule transcriptomic signature; and KIRP originates from cells with a more distal-tubule and connecting-segment profile [39,40]. In our developmental immunofluorescence series, VDR expression undergoes a coordinated redistribution between 10 and 22 weeks of gestation, shifting away from immature glomeruli and toward the maturing proximal and distal convoluted tubules. By the postnatal stage, the strongest VDR signal is concentrated in the proximal and distal tubular segments—the very compartments from which KIRC and KIRP arise. Adult VDR expression, therefore, retains a memory of the maturational program of the cell of origin, and the subtype-specific direction of *VDR* dysregulation in renal carcinoma (loss in collecting-duct-derived KICH; gain in proximal/distal-tubule-derived KIRC and KIRP) can be reconciled with a developmental framework in which the same pathway is reactivated, reprogrammed, or suppressed according to the differentiation state of the transformed cell. We interpret this as a hypothesis to be tested by subtype-stratified *VDR*-target-gene signature analysis, rather than as a definitive mechanistic claim.

Beyond the gene-wise comparisons, the metabolic balance between vitamin D activation (*CYP27B1*) and inactivation (*CYP24A1*) is captured by the log_2_(*CYP24A1/CYP27B1*) ratio. This post hoc exploratory analysis shows a significant decrease in the ratio in KICH (Δ = −1.51; q = 3.5 × 10^−8^) and a smaller but significant decrease in KIRP (Δ = −0.77; q = 0.003); KIRC, in which both enzymes are reduced in parallel, shows no shift in the ratio; BLCA likewise shows no shift. In KICH, the ratio shift is driven principally by the large loss of *CYP24A1*, whereas in KIRP, both arms contribute. The direction of the ratio shift in KICH and KIRP is the opposite of what was reported by Urbschat et al. [23] for clear-cell RCC, in which *CYP24A1* was reported to exceed *CYP27B1*; our data confirm *CYP24A1* reduction in KIRC, but, in this cohort, the parallel reduction of *CYP27B1* leaves the ratio essentially unchanged. The apparent discrepancy with Urbschat et al. likely reflects differences in cohort composition, bulk-tissue versus tumor-microdissection sampling, and the use of normalized RNA-seq versus the qPCR readout of the earlier study. Because the ratio analysis was post hoc, we interpret these findings as hypothesis-generating; they nonetheless point to subtype-specific reprogramming of the vitamin D activation–inactivation balance and provide a quantitative starting point for follow-up.

An important caveat to the TCGA findings is that altered *CYP27B1* and *CYP24A1* expression in tumor samples may not reflect tumor-cell-autonomous transcriptional reprogramming alone. The reciprocal regulation of these two enzymes by parathyroid hormone (PTH)—which induces *CYP27B1* and suppresses *CYP24A1*—and by fibroblast growth factor 23 (FGF23), which exerts the opposite effects via the FGFR1–Klotho complex, is well established and is mediated by kidney-specific enhancer modules acting on both genes [41,42]. Patients with renal carcinoma frequently exhibit elevated circulating FGF23, secondary hyperparathyroidism, and disturbed serum calcium and phosphate, and any of these systemic factors can secondarily drive *CYP27B1* and *CYP24A1* transcription independently of intrinsic tumor reprogramming [43]. The reduction of *CYP24A1* we observe across all three renal cohorts is therefore directionally opposite to what FGF23 elevation alone would predict, suggesting that the observed pattern is unlikely to be a passive systemic response and is more consistent with active tumor-cell or microenvironmental reprogramming of the vitamin D catabolic axis. Definitive resolution of this question requires matched serum mineral and hormone data alongside tumor expression and, ideally, single-cell resolution to localize the relevant transcriptional changes to specific cell populations within the tumor microenvironment.

Several limitations of this study should be acknowledged. Three biological specimens per developmental stage were included, which is consistent with the practical constraints of working with archival human fetal kidney tissue but limits statistical power. Significance is reported at both the field-level (18 fields per stage) and the specimen-level (*n* = 3 per stage); the principal pairwise differences observed at the field level remained significant at the specimen level for 10w-versus-22w, 10w-versus-38w, and 10w-versus-1.5y comparisons in VDR, but smaller pairwise differences became non-significant under specimen-level analysis, as expected at this sample size. Fetal tissues were obtained from spontaneous pregnancy losses or ectopic pregnancies because elective termination is legally restricted in our jurisdiction. Every specimen was screened by H&E histopathology and showed no maceration or overt dysmorphology; however, subclinical inflammation, hormonal dysregulation, or unrecognized genetic factors cannot be fully excluded.

All inferences are based on immunofluorescence; Western blot validation was not feasible because the archival material is exclusively FFPE. Two negative-control strategies (isotype-matched and secondary-only) were used to confirm signal specificity, but mass spectrometry or Western-based confirmation in matched lysates remains a priority for follow-up work. Identification of immature glomeruli, PCT, DCT, and collecting ducts was based on morphology alone, supported by joint review by two senior renal histopathologists. Definitive segment-specific lineage markers (LTL/megalin/AQP2/WT1/synaptopodin) were not co-stained; compartment assignments are therefore subject to interpretive variability, particularly at early developmental stages. *CYP24A1*, the inactivating 24-hydroxylase whose loss-of-function mutations cause idiopathic infantile hypercalcemia, was analysed only at the transcript level (TCGA) and not by immunofluorescence; its spatial distribution during human nephrogenesis therefore remains uncharacterized. Full CYP24A1 protein characterization across the developmental series is the highest-priority planned extension of this work.

“Solid tissue normal” represents a tumor-adjacent rather than truly healthy kidney, and cross-sectional TCGA-based comparisons cannot distinguish primary tumor-driven dysregulation from secondary effects of cancer-associated changes in PTH, FGF23, calcium, and phosphate. Full harmonized comparison with GTEx kidney cortex (via UCSC Xena toil-recompute pipelines) and integration with single-cell fetal-to-adult atlases (e.g., Lake et al., [44]) would refine cell-type-specific interpretations and is identified as the highest-priority bioinformatic follow-up.

The field-level statistical analysis treats non-independent cortical fields as independent observations and is therefore vulnerable to pseudoreplication; we have mitigated this by reporting the specimen-level analysis in parallel and by displaying both in Figure 2e,f.

Building on the present descriptive framework, follow-up work should: extend the cohort to larger numbers of biological replicates, including elective-termination tissues where legally accessible, to confirm the spatiotemporal patterns reported here; include CYP24A1 immunofluorescence on the developmental series to complete the map of the vitamin D activation–inactivation balance—currently planned as a dedicated standalone study with proper antibody optimization on FFPE fetal tissue; perform co-staining with segment-specific markers to enable precise sub-compartment quantification; integrate the developmental data with healthy adult kidney expression resources (GTEx) and single-cell fetal-to-adult atlases such as Lake et al. [44] using harmonized toil-recompute pipelines, to provide a genuine fetal-to-adult comparison at cell-type resolution; measure circulating and tissue vitamin D metabolites in matched specimens to relate local protein expression to metabolic activity; and test, in animal models or organoid systems, whether targeted perturbation of VDR or CYP27B1 during nephrogenesis predisposes to CAKUT-like phenotypes or alters susceptibility to renal injury in adulthood.

## 5. Conclusions

We provide an initial immunofluorescence-based spatial description of VDR and 1α-hydroxylase protein expression across human kidney development. Both proteins are most strongly expressed at 10 gestational weeks and undergo a coordinated redistribution toward proximal and distal convoluted tubules in mid-to-late gestation and postnatal life, paralleling nephron segment maturation. VDR and 1α-hydroxylase co-localize in most nephron compartments at most stages, with a transient dissociation in 22-week glomeruli, consistent with stage-specific autocrine or paracrine vitamin D signaling. Complementary TCGA analysis demonstrates simultaneous dysregulation of VDR, CYP27B1 and CYP24A1 in renal carcinomas (KICH, KIRC, KIRP) but selective dysregulation of CYP27B1 only in bladder urothelial carcinoma, indicating that the developmentally engaged vitamin D pathway retains kidney-specific functional significance in adult renal pathology.

## Figures and Tables

**Table 1:** Primary and secondary antibodies used in the study.

Antibodies		Catalog Number	Host	Dilution	Source
Primary	Vitamin D Receptor/VDR Antibody (D-6)	sc-13133	Mouse	1:50	Santa Cruz Biotechnology Inc., Santa Cruz, CA, USA
	25-hydroxyvitamin D 1a-hydroxylase antibody	PC290	Sheep	1:100	The Binding Site Group Ltd. (Birmingham, UK)
Secondary	Alexa Fluor^®^ 488 AffiniPure^®^ Donkey Anti-Sheep IgG (H + L)	713-545-003	Donkey	1:400	Jackson Immuno Research Laboratories, Inc., West Grove, PA, USA
	Alexa Fluor^®^ 488 AffiniPure™ Donkey Anti-Mouse IgG (H + L)	715-545-150	Donkey	1:400	Jackson Immuno Research Laboratories, Inc., West Grove, PA, USA

**Table 2:** Staining intensity of VDR and VD 1A hydroxylase.

Week/Year	Structure	VDR	1α-Hydroxylase
10 weeks	metanephric cup	++	++
	immature glomerulus	+++	+
	collecting tubule	+	+
22 weeks	glomerulus	+	++
	proximal convoluted tubule	++	++
	distal convoluted tubule	++	++
38 weeks	glomerulus	+	++
	proximal convoluted tubule	++	+
	distal convoluted tubule	+	++
1.5 year	glomerulus	+	+
	proximal convoluted tubule	++	+
	distal convoluted tubule	++	+

## Data Availability

The original contributions presented in this study are included in the article. Further inquiries can be directed to the corresponding author.

## Supplementary Materials

The following supporting information can be downloaded at: https://www.mdpi.com/article/10.3390/medicina62061074/s1.

## Abbreviations

The following abbreviations are used in this manuscript:

1α,25(OH)2D3	1 alpha,25-dihydroxyvitamin D3
1α-hydroxylase	1 alpha-hydroxylase
25(OH)D3	25-hydroxyvitamin D3
ANOVA	Analysis of variance
BLCA	Bladder urothelial carcinoma
CAKUT	Congenital anomalies of the kidney and urinary tract
CI	confidence interval
CYP24A1	Cytochrome P450 family 24 subfamily A member 1 (vitamin D 24-hydroxylase gene)
CYP27B1	Cytochrome P450 family 27 subfamily B member 1 (25-hydroxyvitamin D 1α-hydroxylase gene)
DAPI	4′,6-diamidino-2-phenylindole
dct	Distal convoluted tubule
FDR	false discovery rate
FFPE	Formalin-fixed paraffin-embedded
FGF23	Fibroblast growth factor 23
GTEx	Genotype-Tissue Expression project
H&E	Hematoxylin and eosin staining
HSD	Honestly significant difference
KICH	Kidney chromophobe carcinoma
KIRC	Kidney renal clear cell carcinoma
KIRP	Kidney renal papillary cell carcinoma
MET	Mesenchymal-to-epithelial transition
NR	Nuclear receptor
NR1I1	Nuclear receptor subfamily 1 group I member 1 (alternative name for VDR)
PBS	Phosphate-buffered saline
pct	Proximal convoluted tubule
PTH	Parathyroid hormone
qPCR	Quantitative Polymerase Chain Reaction
RCC	Renal cell carcinoma
RNA	Ribonucleic acid
SD	Standard deviation
STN	Solid tissue normal
TCGA	The Cancer Genome Atlas
TPM	Transcripts per million
ub	Ureteric bud
VDR	Vitamin D receptor

## References

[1] Kato T., Mizuno S. (2017). Nephron, Wilms’ tumor-1 (WT1), and synaptopodin expression in developing podocytes of mice. Exp. Anim..

[2] Little M.H., McMahon A.P. (2012). Mammalian kidney development: Principles, progress, and projections. Cold Spring Harb. Perspect. Biol..

[3] Costantini F., Kopan R. (2010). Patterning a complex organ: Branching morphogenesis and nephron segmentation in kidney development. Dev. Cell.

[4] Lechner M.S., Dressler G.R. (1997). The molecular basis of embryonic kidney development. Mech. Dev..

[5] Saxén L. (1987). Organogenesis of the Kidney.

[6] DeLuca H.F. (2004). Overview of general physiologic features and functions of vitamin D. Am. J. Clin. Nutr..

[7] Holick M.F. (2004). Sunlight and vitamin D for bone health and prevention of autoimmune diseases, cancers, and cardiovascular disease. Am. J. Clin. Nutr..

[8] Ekström L., Storbjörk L., Björkhem-Bergman L. (2016). Genetic Expression Profile of Vitamin D Metabolizing Enzymes in the First Trimester. Horm. Metab. Res..

[9] Hochane M., van den Berg P.R., Fan X., Bérenger-Currias N., Adegeest E., Bialecka M., Nieveen M., Menschaart M., Chuva de Sousa Lopes S.M., Semrau S. (2019). Single-cell transcriptomics reveals gene expression dynamics of human fetal kidney development. PLoS Biol..

[10] Pike J.W., Meyer M.B. (2010). The vitamin D receptor: New paradigms for the regulation of gene expression by 1,25-dihydroxyvitamin D(3). Endocrinol. Metab. Clin. N. Am..

[11] Powala A., Zolek T., Brown G., Kutner A. (2024). Structure and the Anticancer Activity of Vitamin D Receptor Agonists. Int. J. Mol. Sci..

[12] Rochel N. (2022). Vitamin D and Its Receptor from a Structural Perspective. Nutrients.

[13] Goltzman D. (2018). Functions of vitamin D in bone. Histochem. Cell Biol..

[14] Yang S., Li A., Wang J., Liu J., Han Y., Zhang W., Li Y.C., Zhang H. (2018). Vitamin D Receptor: A Novel Therapeutic Target for Kidney Diseases. Curr. Med. Chem..

[15] Kato S., Takeyama K., Kitanaka S., Murayama A., Sekine K., Yoshizawa T. (1999). In vivo function of VDR in gene expression-VDR knock-out mice. J. Steroid Biochem. Mol. Biol..

[16] Jones G., Prosser D.E., Kaufmann M. (2014). Cytochrome P450-mediated metabolism of vitamin D. J. Lipid Res..

[17] Christakos S., Dhawan P., Verstuyf A., Verlinden L., Carmeliet G. (2016). Vitamin D: Metabolism, Molecular Mechanism of Action, and Pleiotropic Effects. Physiol. Rev..

[18] Wagner K.D., Wagner N., Sukhatme V.P., Scholz H. (2001). Activation of vitamin D receptor by the Wilms’ tumor gene product mediates apoptosis of renal cells. J. Am. Soc. Nephrol. JASN.

[19] Flanagan J.N., Wang L., Tangpricha V., Reichrath J., Chen T.C., Holick M.F., Reichrath J., Tilgen W., Friedrich M. (2003). Regulation of the 25-hydroxyvitamin D-1alpha-hydroxylase gene and its splice variant. Vitamin D Analogs in Cancer Prevention and Therapy. Recent Results in Cancer Research.

[20] Hewison M., Burke F., Evans K.N., Lammas D.A., Sansom D.M., Liu P., Modlin R.L., Adams J.S. (2007). Extra-renal 25-hydroxyvitamin D3-1alpha-hydroxylase in human health and disease. J. Steroid Biochem. Mol. Biol..

[21] Schlingmann K.P., Cassar W., Konrad M. (2018). Juvenile onset IIH and CYP24A1 mutations. Bone Rep..

[22] Schlingmann K.P., Kaufmann M., Weber S., Irwin A., Goos C., John U., Misselwitz J., Klaus G., Kuwertz-Bröking E., Fehrenbach H. (2011). Mutations in CYP24A1 and idiopathic infantile hypercalcemia. N. Engl. J. Med..

[23] Urbschat A., Paulus P., von Quernheim Q.F., Brück P., Badenhoop K., Zeuzem S., Ramos-Lopez E. (2013). Vitamin D hydroxylases CYP2R1, CYP27B1 and CYP24A1 in renal cell carcinoma. Eur. J. Clin. Investig..

[24] Kelam J., Kelam N., Filipović N., Komić L., Racetin A., Komić D., Kostić S., Kuzmić Prusac I., Vukojević K. (2024). Expression of Congenital Anomalies of the Kidney and Urinary Tract (CAKUT) Candidate Genes EDA2R, PCDH9, and TRAF7 in Normal Human Kidney Development and CAKUT. Genes.

[25] Kelam N., Ogorevc M., Gotovac I., Kuzmić Prusac I., Vukojević K., Saraga-Babić M., Mardešić S. (2025). Analysis of Kallikrein 6, Acetyl-α-Tubulin, and Aquaporin 1 and 2 Expression Patterns During Normal Human Nephrogenesis and in Congenital Anomalies of the Kidney and Urinary Tract (CAKUT). Genes.

[26] Kelam N., Racetin A., Polović M., Benzon B., Ogorevc M., Vukojević K., Glavina Durdov M., Dunatov Huljev A., Kuzmić Prusac I., Čarić D. (2022). Aberrations in FGFR1, FGFR2, and RIP5 Expression in Human Congenital Anomalies of the Kidney and Urinary Tract (CAKUT). Int. J. Mol. Sci..

[27] Maglica M., Kelam N., Perutina I., Racetin A., Rizikalo A., Filipović N., Kuzmić Prusac I., Mišković J., Vukojević K. (2024). Immunoexpression Pattern of Autophagy-Related Proteins in Human Congenital Anomalies of the Kidney and Urinary Tract. Int. J. Mol. Sci..

[28] Pavic B., Ogorevc M., Boric K., Vukovic D., Saraga-Babic M., Mardesic S. (2023). Connexin 37, 40, 43 and Pannexin 1 Expression in the Gastric Mucosa of Patients with Systemic Sclerosis. Biomedicines.

[29] Pavlović N., Kelam N., Racetin A., Filipović N., Pogorelić Z., Prusac I.K., Vukojević K. (2024). Expression Profiles of ITGA8 and VANGL2 Are Altered in Congenital Anomalies of the Kidney and Urinary Tract (CAKUT). Molecules.

[30] Cicchetti D. (1994). Guidlines, Criteria, and Rules of Thumb for Evaluating Normed and Standardized Assessment Instrument in Psychology. Psychol. Assess..

[31] Yamagata M., Kimoto A., Michigami T., Nakayama M., Ozono K. (2001). Hydroxylases involved in vitamin D metabolism are differentially expressed in murine embryonic kidney: Application of whole mount in situ hybridization. Endocrinology.

[32] Arora J., Froelich N.E., Tang M., Weaver V., Paulson R.F., Cantorna M.T. (2024). Developmental Vitamin D Deficiency and the Vitamin D Receptor Control Hematopoiesis. J. Immunol..

[33] Jelcic D., Puzovic V., Benzon B., Palada I., Jerkovic J., Vulic M. (2023). Immunohistochemical Expression of Placental Vitamin D Receptors in Pregnancies Complicated by Early and Late-Onset Preeclampsia. Acta Medica Okayama.

[34] Song Y.S., Jamali N., Sorenson C.M., Sheibani N. (2023). Vitamin D Receptor Expression Limits the Angiogenic and Inflammatory Properties of Retinal Endothelial Cells. Cells.

[35] Zehnder D., Bland R., Chana R.S., Wheeler D.C., Howie A.J., Williams M.C., Stewart P.M., Hewison M. (2002). Synthesis of 1,25-dihydroxyvitamin D(3) by human endothelial cells is regulated by inflammatory cytokines: A novel autocrine determinant of vascular cell adhesion. J. Am. Soc. Nephrol. JASN.

[36] Fischer D., Thome M., Becker S., Cordes T., Diedrich K., Friedrich M., Thill M. (2009). 25-Hydroxyvitamin D3 1alpha-hydroxylase splice variants in benign and malignant ovarian cell lines and tissue. Anticancer. Res..

[37] Wang Y., Zhu J., DeLuca H.F. (2015). The vitamin D receptor in the proximal renal tubule is a key regulator of serum 1alpha,25-dihydroxyvitamin D(3). Am. J. Physiol. Endocrinol. Metab..

[38] Xia X., Xu F., Dai D., Xiong A., Sun R., Ling Y., Qiu L., Wang R., Ding Y., Lin M. (2024). VDR is a potential prognostic biomarker and positively correlated with immune infiltration: A comprehensive pan-cancer analysis with experimental verification. Biosci. Rep..

[39] Davis C.F., Ricketts C.J., Wang M., Yang L., Cherniack A.D., Shen H., Buhay C., Kang H., Kim S.C., Fahey C.C. (2014). The somatic genomic landscape of chromophobe renal cell carcinoma. Cancer Cell.

[40] Ricketts C.J., De Cubas A.A., Fan H., Smith C.C., Lang M., Reznik E., Bowlby R., Gibb E.A., Akbani R., Beroukhim R. (2018). The Cancer Genome Atlas Comprehensive Molecular Characterization of Renal Cell Carcinoma. Cell Rep..

[41] Bär L., Stournaras C., Lang F., Föller M. (2019). Regulation of fibroblast growth factor 23 (FGF23) in health and disease. FEBS Lett..

[42] Meyer M.B., Pike J.W. (2020). Mechanistic homeostasis of vitamin D metabolism in the kidney through reciprocal modulation of Cyp27b1 and Cyp24a1 expression. J. Steroid Biochem. Mol. Biol..

[43] Edmonston D., Wolf M. (2020). FGF23 at the crossroads of phosphate, iron economy and erythropoiesis. Nat. Rev. Nephrol..

[44] Lake B.B., Menon R., Winfree S., Hu Q., Melo Ferreira R., Kalhor K., Barwinska D., Otto E.A., Ferkowicz M., Diep D. (2023). An atlas of healthy and injured cell states and niches in the human kidney. Nature.

