# Supplementary material for: Vitamin D Signaling from Nephrogenesis to Neoplasia: Spatial Protein Expression in Fetal Kidney and Transcriptomic Dysregulation in Renal Tumors
Source: Medicina (Kaunas). 2026 Jun 1;62(6):1074. doi: 10.3390/medicina62061074 (PMC13303805; doi:10.3390/medicina62061074)
Supplement: Supplementary file 1 [file medicina-62-01074-s001.zip › medicina-4223906-supplementary.pdf]

medicina

MDPI

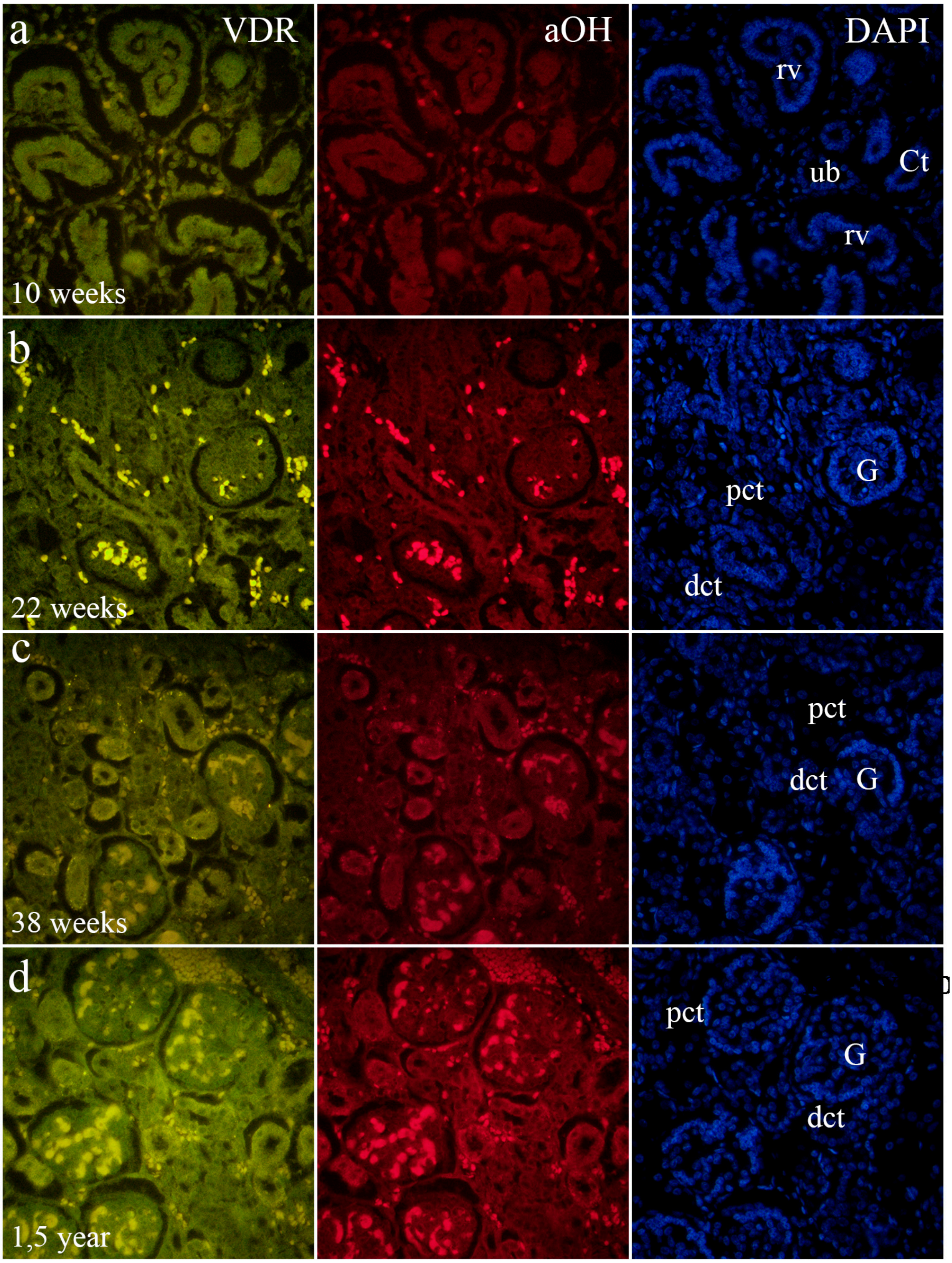

Supplementary Figure S1. Secondary-only negative controls across developmental stages, confirming target-specific signal in primary-stained sections. Representative cortical fields are shown at (a) 10 gestational weeks, (b) 22 gestational weeks, (c) 38 gestational weeks, and (d) 1.5 years postnatal. Adjacent paraffin sections were processed using the identical protocol described in Methods 2.2

(deparaffinisation, citrate-buffer antigen retrieval, blocking, washes) except that primary antibodies (anti-VDR D-6 clone and anti-1α-hydroxylase PC290) were omitted; sections received only the corresponding Alexa Fluor 488–conjugated secondary antibodies (donkey anti-mouse IgG and donkey anti-sheep IgG; Jackson ImmunoResearch Laboratories, West Grove, PA, USA) at the same dilution (1:400) and incubation time (1 h, humidified chamber) used in the primary stainings. Coverslips were mounted with DAPI to verify tissue orientation. Image acquisition was performed on the same Olympus BX51 epifluorescence microscope (Nikon DS-Ri2 camera) under identical exposure time, gain and dynamic range as the corresponding primary-stained micrographs presented in Figure 2 (×40 magnification). In all four developmental stages, the secondary-only fields show only weak diffuse background and a small amount of structural autofluorescence — most pronounced in the proximal tubular brush border and in red blood cells within glomerular and peritubular capillary lumina, both of which are well-documented autofluorescence sources in fixed renal tissue. This background was used to calibrate the ImageJ thresholding step described in Methods 2.3 and is below the segmentation threshold applied to the primary-stained images. Together with the parallel isotype-matched controls described in Methods 2.2, these images demonstrate that the cytoplasmic and structure-specific signal reported in Figure 2 is attributable to the primary antibodies rather than to non-specific secondary binding or to tissue autofluorescence. Scale bar = 100 μm (applies to all panels).

Supplementary Table S1. Individual per-field fluorescence-area measurements for VDR and 1α-hydroxylase across human kidney developmental stages. For each of the 12 specimens (3 biological replicates per developmental stage: 10 gestational weeks, 22 gestational weeks, 38 gestational weeks, and 1.5 years postnatal), three non-adjacent paraffin sections were immunostained and six non-overlapping cortical fields per specimen were imaged at ×40 magnification, yielding 18 individual field measurements per developmental stage. Values are the percentage of fluorescence-positive area in each ×40 cortical field, quantified in ImageJ as described in Methods 2.3 (red-channel subtraction, 8-px median-filter background subtraction, triangle auto-threshold, analyze-particles). Summary statistics at the bottom of the table reproduce the mean ± SD values plotted in Figure 2e,f and reported in the Abstract. Specimen identifiers (S1, S2, S3) within each stage refer to independent biological replicates; field identifiers (F1–F6) refer to non-overlapping cortical fields imaged within each specimen. Underlying data for all statistical comparisons reported in Section 3.2 and in the Figure 2 legend (field-level and specimen-level Tukey HSD post-hoc tests on one-way ANOVA).

Stage	Specimen	Field	VDR area %	1α-OH area %
10w	S1	F1	3.806	5.475
10w	S1	F2	3.403	5.322
10w	S1	F3	3.940	5.147
10w	S1	F4	3.997	5.003
10w	S1	F5	3.130	4.876
10w	S1	F6	3.324	5.258
10w	S2	F1	3.158	5.398
10w	S2	F2	3.025	5.386
10w	S2	F3	3.115	5.516
10w	S2	F4	2.864	5.550
10w	S2	F5	3.384	5.105
10w	S2	F6	3.353	5.745
10w	S3	F1	3.459	5.251
10w	S3	F2	3.778	6.028
10w	S3	F3	3.580	5.774
10w	S3	F4	3.182	5.562
10w	S3	F5	3.550	5.925
10w	S3	F6	3.152	5.660
22w	S1	F1	0.657	2.778
22w	S1	F2	0.582	2.574

22w	S1	F3	0.571	2.920
22w	S1	F4	0.532	2.908
22w	S1	F5	0.684	2.219
22w	S1	F6	0.574	2.322
22w	S2	F1	0.653	3.426
22w	S2	F2	0.659	3.232
22w	S2	F3	0.730	2.506
22w	S2	F4	0.716	4.015
22w	S2	F5	0.720	2.544
22w	S2	F6	0.722	2.577
22w	S3	F1	0.923	2.966
22w	S3	F2	0.719	3.611
22w	S3	F3	0.710	4.042
22w	S3	F4	0.686	3.331
22w	S3	F5	0.801	3.276
22w	S3	F6	0.842	3.474
38w	S1	F1	2.794	2.816
38w	S1	F2	2.140	3.355
38w	S1	F3	2.154	1.888
38w	S1	F4	3.482	3.782
38w	S1	F5	3.565	3.727
38w	S1	F6	3.385	0.333
38w	S2	F1	1.351	4.334
38w	S2	F2	2.159	4.662
38w	S2	F3	2.055	5.140
38w	S2	F4	2.147	3.487
38w	S2	F5	2.735	2.873
38w	S2	F6	2.152	3.325
38w	S3	F1	2.678	6.229
38w	S3	F2	2.127	4.550
38w	S3	F3	2.327	4.655
38w	S3	F4	2.635	6.944
38w	S3	F5	0.755	4.628
38w	S3	F6	1.779	4.493
1.5y	S1	F1	1.209	3.518
1.5y	S1	F2	1.189	3.852
1.5y	S1	F3	1.233	4.816
1.5y	S1	F4	1.445	2.612
1.5y	S1	F5	1.162	1.820
1.5y	S1	F6	1.382	3.482
1.5y	S2	F1	1.043	5.125
1.5y	S2	F2	1.205	2.681
1.5y	S2	F3	1.265	3.825
1.5y	S2	F4	1.316	4.737
1.5y	S2	F5	1.331	4.651
1.5y	S2	F6	1.340	4.481
1.5y	S3	F1	1.429	5.022

https://doi.org/10.3390/medicina62061074

1.5y	S3	F2	1.416	4.398
1.5y	S3	F3	1.574	4.250
1.5y	S3	F4	1.448	6.647
1.5y	S3	F5	1.318	4.033
1.5y	S3	F6	1.335	6.730
**Stage**	**n (fields)**		**VDR mean ± SD**	**1α-OH mean ± SD**
10w	18		3.40 ± 0.32	5.44 ± 0.31
22w	18		0.69 ± 0.10	3.04 ± 0.54
38w	18		2.36 ± 0.71	3.96 ± 1.51
1.5y	18		1.31 ± 0.13	4.26 ± 1.25

Each value is the percentage of fluorescence-positive area in a single ×40 cortical field, quantified in ImageJ as described in Methods 2.3. Six non-overlapping fields were imaged per specimen; three specimens were analysed per developmental stage; total 18 fields per stage. Summary statistics at the bottom of the table reproduce the values plotted in Figure 2e,f.

https://doi.org/10.3390/medicina62061074

Supplementary Table S2. TCGA tumor-versus-normal comparisons of *VDR*, *CYP27B1* and *CYP24A1* expression, with Benjamini–Hochberg false discovery rate correction. Section A reports the 12 pre-specified gene-wise comparisons of mean TPM expression in tumor versus solid-tissue-normal samples for *VDR*, CYP27B1 and *CYP24A1* across four TCGA cohorts: kidney chromophobe carcinoma (KICH), kidney renal clear cell carcinoma (KIRC), kidney renal papillary cell carcinoma (KIRP), and bladder urothelial carcinoma (BLCA). Section B reports the 4 post-hoc exploratory $\log_2((CYP24A1 + 1)/(CYP27B1 + 1))$ ratio comparisons in the same four cohorts. All 16 comparisons were performed using unpaired two-tailed Welch's t-tests and were jointly corrected for multiple testing using the Benjamini–Hochberg false discovery rate procedure as a single comparison family. n (normal) and n (tumor): number of solid-tissue-normal and tumor samples, respectively. Mean N, Mean T: mean $\log_2$-transformed TPM (gene-wise rows) or mean $\log_2$-ratio (ratio rows). $\log_2$FC: $\log_2$-fold change between tumor and normal. p (raw): unadjusted p-value from Welch's t-test. q (BH): Benjamini–Hochberg-adjusted q-value. Significance codes: **** q < 0.0001, *** q < 0.001, ** q < 0.01, * q < 0.05, ns = not significant. All 12 gene-wise comparisons reaching nominal significance at raw p < 0.05 retained significance after BH correction.

Comparison	n (normal)	n (tumor)	Mean N	Mean T	$\log_2$FC	p (raw)	q (BH)	Sig.
A. Pre-specified gene-wise comparisons (n = 12)								
VDR (KICH)	25	66	6.47	2.65	−1.03	<0.0001	2×10^{-4}	***
VDR (KIRC)	72	533	4.79	7.75	+0.59	<0.0001	2×10^{-4}	***
VDR (KIRP)	32	290	4.04	7.37	+0.73	<0.0001	2×10^{-4}	***
VDR (BLCA)	19	408	6.97	7.00	+0.01	0.97	0.97	ns
CYP27B1 (KICH)	25	66	5.79	6.73	+0.19	0.026	0.035	*
CYP27B1 (KIRC)	72	533	5.45	4.15	−0.32	<0.0001	2×10^{-4}	***
CYP27B1 (KIRP)	32	290	5.09	5.87	+0.17	0.020	0.029	*
CYP27B1 (BLCA)	19	408	3.66	6.17	+0.62	<0.0001	2×10^{-4}	***
CYP24A1 (KICH)	25	66	6.88	3.09	−0.95	<0.0001	2×10^{-4}	***
CYP24A1 (KIRC)	72	533	6.03	4.66	−0.31	<0.0001	2×10^{-4}	***
CYP24A1 (KIRP)	32	290	6.22	4.91	−0.29	0.020	0.029	*
CYP24A1 (BLCA)	19	408	3.95	4.93	+0.26	0.19	0.24	ns
B. Post-hoc $\log_2$(*CYP24A1*/*CYP27B1*) ratio comparisons (n = 4)								
$\log_2$ ratio (KICH)	25	66	+0.27	−1.24	−1.51	2×10^{-9}	3.5×10^{-8}	****
$\log_2$ ratio (KIRC)	72	533	+0.09	−0.03	−0.12	0.42	0.44	ns
$\log_2$ ratio (KIRP)	32	290	+0.35	−0.42	−0.77	0.0015	0.003	**
$\log_2$ ratio (BLCA)	19	408	−0.28	−0.52	−0.24	0.41	0.44	ns

Tumor-versus-normal comparisons by unpaired two-tailed Welch's t-test on TPM values (gene-wise) or per-sample $\log_2((CYP24A1{+}1)/(CYP27B1{+}1))$ ratios (ratio rows). Benjamini–Hochberg FDR correction applied across all 16 comparisons (sections A + B) as a single comparison family. Significance codes: **** q < 0.0001, *** q < 0.001, ** q < 0.01, * q < 0.05, ns = not significant. Green shading indicates significance after BH FDR correction. All 12 comparisons reaching nominal significance at raw p < 0.05 retained significance after BH correction.

https://doi.org/10.3390/medicina62061074
